# Weighted Single-Step GWAS Reveals Genomic Regions Associated with Female Fertility in the Spanish Retinta Beef Cattle

**DOI:** 10.3390/ani15182665

**Published:** 2025-09-11

**Authors:** Rosa María Morales, Gabriel Anaya Calvo-Rubio, Chiraz Ziadi, María Ángeles Vargas-Pérez, Sebastián Demyda-Peyrás, Antonio Molina

**Affiliations:** Department of Genetics, University of Córdoba, 14071 Córdoba, Spain; v22mocir@uco.es (R.M.M.); b22ancag@uco.es (G.A.C.-R.); z42vapem@uco.es (M.Á.V.-P.); ge2depes@uco.es (S.D.-P.);

**Keywords:** female fertility traits, wssGBLUP, GWAS, Retinta beef cattle

## Abstract

Improving reproductive efficiency in beef cattle remains a key objective for sustainable genetic progress, particularly in extensively managed autochthonous breeds such as the Spanish Retinta. In this study, we applied a weighted single-step genome-wide association approach (wssGWAS) to identify genomic regions associated with four fertility-related traits: age at first calving (AFC), interval between first and second calving (IC12), average calving interval (ACI), and reproductive efficiency (RE). A total of 215,125 calving records from 44,032 cows and the genomic information of 1030 animals (Axiom™ Bovine Genotyping v3 Array 65k) were analyzed. Heritability was estimated using a single-step genomic best linear unbiased prediction (ssGBLUP) that incorporated both pedigree and genomic data, and estimates ranged from 0.15 (0.008) for AFC to 0.27 (0.012) for ACI. The wssGWAS identified 96 1 Mb-windows explaining over 1% of additive genetic variance (40 of them are common for more than one trait and 46 windows are unique), notably on chromosomes 2 and 5. Candidate genes related to folliculogenesis, steroidogenesis, immune modulation, and cell cycle control were identified, including *ACVR1B*, *AMHR2*, *CYP27B1*, *CDK2*, and *IFNG*. Additionally, a significant proportion of lncRNAs were detected, suggesting regulatory roles in reproductive processes through the modulation of gene expression at different levels. These findings enhance our understanding of the genetic architecture underlying female fertility in beef cattle and provide valuable markers for incorporation into genomic selection programs aimed at improving reproductive performance and long-term sustainability in the Retinta breed.

## Simple Summary

Reproductive efficiency in beef cattle remains a limiting factor for productivity, particularly in autochthonous breeds managed under extensive conditions, where traditional phenotypic selection is hindered by low heritability and strong environmental influence. This study addresses the challenge of improving female fertility in the Spanish Retinta breed by applying a weighted single-step genome-wide association study (wssGWAS) that integrates pedigree, phenotypic, and genomic information. Using a comprehensive dataset of the reproductive history of 44,000 cows (over 215,000 calving records) and genomic information of 1030 animals (65K), the analysis identified multiple genomic regions significantly associated with key fertility traits, including age at first calving (AFC), interval between first and second calving (IC12), average calving interval (ACI), and reproductive efficiency (RE). Notably, several candidate genes involved in ovarian function, cell cycle regulation, and hormonal signaling, along with a substantial proportion of lncRNAs, were detected. These findings provide new insights into the genetic architecture of fertility under extensive production systems and offer practical tools for enhancing selection accuracy. By incorporating these genomic markers into breeding programs, it is possible to accelerate genetic progress, improve reproductive performance, and contribute to the long-term sustainability of the Retinta breed.

## 1. Introduction

The beef cattle industry constitutes approximately 6.5% of the total value of Spain’s final agricultural production and accounts for 16.4% of the final livestock output, generating nearly €4.1 billion in economic value in 2022 [1]. This sector is predominantly based in rural regions, where it plays a pivotal role in sustaining employment and curbing rural depopulation, particularly in areas with limited economic alternatives. Moreover, beef cattle farming contributes to the preservation of traditional practices and cultural heritage, while simultaneously supporting ecosystem conservation and promoting environmental sustainability [2].

Within this context, the Retinta cattle is an autochthonous Spanish breed adapted to semi-arid conditions and typically managed under extensive systems linked to the Dehesa ecosystem, distinguished by its strong maternal abilities [3,4]. The breed is also found in other countries, such as Portugal, Argentina, and Brazil. According to the National Association of Retinta Breeders (ACRE), the population comprises approximately 250,000 cows, of which only 10% are enrolled in official performance recording and genetic improvement programs. These programs primarily target enhancements in growth and reproductive performance, with a secondary emphasis on carcass quality [5].

The objective measurement of reproductive traits in extensive production systems is inherently challenging due to their high sensitivity to environmental factors and herd management practices, resulting in generally low heritabilities, and consequently limited genetic progress [6]. Moreover, selection for growth traits has been shown to negatively affect reproductive performance, further restricting the efficacy of conventional selection approaches for reproductive traits [7,8]. In this context, the identification of molecular markers linked to female productive potential is critical. Such markers can facilitate the early and accurate estimation of breeding values and expand the selection base, thereby accelerating genetic progress across the population.

Among reproductive traits, fertility holds particular economic significance for beef cattle producers. Given the constraints of extensive systems, indirect indicators that are both informative and practically measurable are essential for identifying females with superior fertility. Two such indicators, calving interval (CI) and age at first calving (AFC), are widely recognized. However, cows with shorter calving intervals are often those that experienced delayed first calving, and selecting for this trait may inadvertently lead to increased age at puberty [9]. Empirical evidence reveals strong correlations between AFC and subsequent calving ages, as well as between calving age and inter-calving intervals. Consequently, a delayed first calving is unlikely to be compensated by shorter intervals in later reproductive cycles [8]. There is general agreement that the optimal beef cow should be precocious and capable of producing one calf per year [10,11]. For the Retinta breed, the ideal AFC is two years, and the optimal CI is one year [12].

These two traits are integrated into a single parameter termed reproductive efficiency (RE), defined as the percentage deviation between the actual number of calvings a cow has at a given age and the number it would be expected to have under optimal conditions. RE has been effectively employed in selection programs for various species, including horses [13], goats [14], and more recently, beef cattle [4]. Notably, this trait has demonstrated greater heritability and reliability than other reproductive traits (h^2^ ≈ 0.30 ± 0.003) in Retinta cattle [4]. As a result, RE has been formally incorporated into ACRE’s breeding program as a principal selection criterion.

Recent advances in genomic technologies have considerably enhanced the ability to identify associations between single nucleotide polymorphisms (SNPs) and phenotypic variation, thereby deepening our understanding of complex traits. Among these technologies, genome-wide association studies (GWAS) have emerged as a cornerstone in contemporary genetic research. In beef cattle, GWAS have been extensively applied to traits such as growth, carcass composition, fatty acid profiles, meat quality, and other production-related characteristics [15,16,17,18,19,20,21]. Nevertheless, GWAS targeting reproductive traits in beef cows remain limited [4,22,23,24,25,26].

Several studies have applied genomic methods to identify variants associated with reproductive traits in beef cattle. For instance, Stegemiller et al. Stegemiller, et al. [25] performed a GWAS on age at first calving (AFC) in beef cattle, identifying relevant genetic variants. Similarly, Carvalho Filho et al. [27] used whole-genome sequencing to map candidate genes associated with AFC and other reproductive traits in Nellore cattle, highlighting the importance of considering additive and dominance effects. However, most of these studies have focused on dairy breeds or intensively managed systems, limiting their applicability to locally adapted breeds reared under extensive conditions. Our study is focus on the Retinta breed, an autochthonous Spanish cattle population adapted to extensive management, and in incorporating reproductive efficiency (RE) as an integrated selection criterion. Most recently, the single-step genomic best linear unbiased prediction (ssGBLUP) method, which integrates pedigree, phenotype, and genotype information into a unified framework, was proposed by Misztal et al. [28]. Nevertheless, Wang et al. [29] reported that ssGBLUP is based on a model that assumes equal variance for SNP effects. This fact limits the use of this method since it does not reflect the real situation for all traits of economic interest. To overcome this limitation, Wang et al. [29] proposed weighted ssGBLUP (WssGBLUP), which combines pedigree, phenotype, and genotype data, with different weights are attributed to the markers in an iterative process to update the SNP solutions. It has proven to be a very efficient method to estimate the contribution of individual SNPs to the genetic variance, improving the detection of genomic regions associated with complex traits. This approach is particularly advantageous in livestock populations, as it accommodates unbalanced data and enables the inclusion of both genotyped and non-genotyped animals, enhancing the accuracy of GWAS analysis.

The present study aims to analyze key reproductive variables used in genetic evaluations, namely age at first calving (AFC), interval between first and second calving (IC12), average calving interval (ACI), and reproductive efficiency (RE), to identify genomic regions accounting for the highest cumulative variance in fertility-related traits in the Retinta breed, and to characterize the associated genes and metabolic pathways.

## 2. Materials and Methods

### 2.1. Ethical Statement

The samples used in this study were provided by the Breeders Association and were obtained during the compulsory sanitary practices by official veterinarians, avoiding the disturbance of the animals only for research purposes. Data were obtained from the official recording program of the National Association of Retinta Breeders.

### 2.2. Data Recording

In this study, we analyzed 285,135 calving records collected by the National Retinta Breeders’ Association. The dataset comprises information on 69,212 cows that produced offspring with 3630 different bulls. Pedigree information from the active cows included 2990 grandsires and 30,430 granddams). After filtering and pruning incomplete and outlying data, 44,032 cows were retained for the genetic analysis. The pedigree was extended to include all the available information in the breed database, with a total of 56,156 animals. The inbreeding coefficients of the cow (Fc) were determined according to the methodology described by Meuwissen and Luo [30] using the optiSel package [31] from the R statistical environment [32]. In addition, we estimated the calving number (Cn) and the herd-year-breeding season combination (HYS) in each observation using self-made R-scripts together with the Tidyverse [33] and data.table [34] packages. We defined the fertility of the cows using the following traits: age at first calving (AFC), interval between first and second calving (IC12), average calving interval (ACI), and reproductive efficiency (RE), calculated as the deviation between the optimal and real parity number of females at each age, as described by Jiménez et al. [4].

### 2.3. Animal Sampling for the Genomic Assays

A total of 1030 controlled cows were selected for the genomic assay based on the following criteria: representation of the highest number of herds (88), low level of kinship, pedigree completeness and having data for the studied parameters.

Blood samples were collected using EDTA-K3 BD vacutainers™ (BD, Madrid, Spain) by the official technicians of ACRE.

### 2.4. Genotyping and Quality Control

Genomic DNA was isolated from blood using the commercial DNA purification kit DNeasy Blood & Tissue Kit (Qiagen, Germantown, MD, USA), following the manufacturer’s protocol. Quantity and quality of the DNA were measured with a Thermo Scientific™ NanoDrop™ One (Thermo Fisher Scientific Inc., Waltham, MA, USA). Those samples with optimal ratios (absorbance ratios of A260/A280 and A260/230 of 1.8 to 2) were genotyped using the Axiom™ Bovine Genotyping v3 Array (Thermo Fisher Scientific Inc., Waltham, MA, USA), including more than 63,000 SNPs. Raw data were processed in the Axiom analysis suite package v5.0 [35] where all the SNPs had the highest quality levels of genotyping results (DQC ≥ 0.82 and individual call rate QC ≥ 0.90). Subsequently, the dataset was pruned keeping the markers from autosomal chromosomes (BTA1 to BTA29). After that, SNPs without correct annotations and those with a minor allele frequency < 0.01 were removed, which left 45,331 variants using PLINK software v1.9 [36].

### 2.5. Weighted Single-Step GREML Method

The significance of the fixed effects for female fertility traits was determined using the ‘GLM2’ package [37] in the R statistical environment v4.4.0 [38]. All the fixed effects were significant at the 0.05 level. 

To analyze female fertility traits, a univariate model was employed as follows:

y=µ+Xb+Za+Wc+e

where y is the vector of phenotypic observations for the corresponding trait; µ is the vector of overall mean with identical elements; b is the vector of fixed effects, including inbreeding as a covariate (except for AFC); age at last calving as a covariate for RE; age at first calving (except for AFC, 3 classes: 1 < 30 months; 2 >= 30 and <36; 3 >= 36); a is the vector of random additive genetic effects; c is the vector of random effect of the interaction herd-year-season of birth of the cow (12,555 classes); e is the vector of random residuals; and X, Z, and W are incidence matrices relating observations to fixed, random additive genetic and random interaction herd-year-season of birth of the cow effects, respectively. It was assumed that $a\sim N(0,H\sigma_{a}^{2}$), hys$\sim N(0,I\sigma_{hys}^{2})$, and $e\sim N\left(0,I\sigma_{e}^{2}\right)$ for all traits, where $\sigma_{a}^{2}$, $\sigma_{hys}^{2},$ and $\sigma_{e}^{2}$ are the additive genetic, herd-year-season of the birth cow, and residual variances, respectively. The matrix H was obtained following Aguilar et al. [39] by combining the numerator relationship matrix (A) with the genomic relationship matrix (G). The inverse of H matrix is:

$$H^{-1}=A^{-1}+\left[\begin{array}{cc} 0 & 0\\ 0 & G^{-1}-A_{22}^{-1} \end{array}\right]$$

where A is the pedigree-based relationship matrix for all animals; A_22_ is the pedigree-based relationship matrix for genotyped animals; and G is the genomic relationship matrix for genotyped animals, obtained following VanRaden [40] as:

$$G=\frac{ZZ^{\prime }}{\sum_{i=1}^{N}2p_{i}\left(1-p_{i}\right)}$$

where Z is a matrix of SNP genotypes; N is the number of SNPs; and p_i_ is the minor allele frequency of *i*-th SNP.

Variance components and genomic estimated breeding values (GEBVs) of the studied traits were estimated using the restricted maximum likelihood approach with BLUPF90+ v2.60 software [41].

In the first iteration, a single-step genomic restricted maximum likelihood (ssGREML) approach was employed using the G matrix. Then, the estimates of SNP effects were obtained by back-solving GEBVs from ssGREML, according to Wang et al. [29]:

$$\hat{a}=DZ^{\prime }{\left(ZDZ^{\prime }\right)}^{-1}{\hat{u}}_{g}$$

where $\hat{a}$ is a vector of SNP effects; D is a diagonal matrix of weights (D is equal to the identity matrix for ssGREML); Z is the centered matrix of SNP genotypes; and ${\hat{u}}_{g}$ is the vector of GEBVs from genotyped animals only.

Estimates of SNP effects were used to estimate the individual variance of each SNP effect [42]:

$$\sigma_{u,i}^{2}=2{\hat{a}}_{i}^{2}p_{i}(1-p_{i})$$

where $p_{i}$ is the minor allele frequency of SNPi. SNP effects and variances were calculated using the POSTGSF90 software [39]. Then, the vector of variances of SNP effects was used as weights in matrix D to construct the weighted matrix G ($G^{*}$) as described in Wang et al. [29]:

$$G^{*}=\frac{ZDZ^{\prime }}{\sum_{i=1}^{N}2p_{i}\left(1-p_{i}\right)}$$

GEBVs were estimated again using the BLUPF90+ software [41] by considering weights for each SNP via the $G^{*}$ matrix included in the H matrix. This process was carried out iteratively, with weights estimated at each iteration as described in Wang et al. [29].

### 2.6. Genome-Wide Association Study Analysis

The GWAS analysis was based on detecting genomic regions of 1 Mb that explained more than 1% of the variability in each trait. The percentage of genetic variance explained by the *i*-th set of SNPs included in a 1 Mb window (*i*-th SNP window) was calculated as described by Wang et al. [29] as:

$$\frac{Var(a_{i})}{\sigma_{a}^{2}}\times \;100\%=\frac{Var\;(\sum_{j=1}^{x}Z_{j}{\hat{u}}_{j})}{\sigma_{a}^{2}}\times \;100\%$$

where $a_{i}$ is the genetic value of the *i*-th SNP window of consecutive SNPs; $\sigma_{a}^{2}$ is the total additive genetic variance; $Z_{j}$ is the vector of gene content of the *j*-th SNP for all individuals; and ${\hat{u}}_{j}$ is the effect of the *j*-th SNP within the *i*-th window.

The GWAS analysis was performed with the POSTGSF90 software [39], using the 1 Mb overlapping windows option. SNP sets explaining more than 1% of the additive genetic variance were selected.

For the ssGREML and wssGREML analyses, all animals in the pedigree were included, whereas for the estimation of SNP effects and GWAS analyses, only genotyped animals were considered.

### 2.7. Identification of Candidate Genes and Gene Network

The windows that GBLUP found to have a high effect were evaluated for the genes located in them based on the *Bos taurus* ARS-UCD2.0 reference sequence. To this purpose, the BioMart application from the Ensembl repository was used. Next, functional and physical associations between proteins encoded by genes common to different traits were predicted using STRING [43], considering experimental evidence, information from databases, and co-expression levels, with a minimum interaction score of 0.4. The network was further analyzed with Cytoscape v3.10.3 [44], where a cluster analysis was performed using MCODE [45] with default parameters.

## 3. Results and Discussion

The results of this study provide a comprehensive overview of the phenotypic, genetic, and genomic factors influencing female fertility in the Retinta breed. The high variability observed allows substantial potential for improvement of these reproductive traits. First, descriptive phenotypic statistics will be described; then we will focus on heritability and variance components and link them to GWAS to finally locate common genes between traits. The genomic analysis identified relevant regions and candidate genes with key biological roles in reproduction, highlighting the value of integrating genomic tools into breeding programs for this locally adapted and economically important cattle population.

### 3.1. Descriptive Phenotypic Statistics

Summary statistics of the traits studied in the Retinta breed are presented in Table 1. The high coefficient of variation stands out in all traits, which is indicative of the wide range of environmental and genetic conditions of the population analyzed, as well as the extensive possibilities for improving these traits in the Retinta breed. Compared to other continental and English European beef breeds, the Retinta shows lower reproductive performance in terms of AFC and ACI. According to data published by Roughsedge et al. [46] and Márton et al. [47], the AFC range for these breeds from 2.62 ± 0.27 to 2.78 ± 0.26 years, while the CI oscillates between 391 ± 63 and 402 ± 58 days, respectively. These differences suggest that Retinta’s reproductive management and productivity could be improved. However, it is important to consider that the breeding and environmental conditions differ greatly among these breeds. In the case of Retinta, the animals are raised under much harsher and less controlled conditions. When focusing on Spanish breeds managed under similar extensive production systems, the results are more comparable. Gutiérrez et al. [8] reported an average CI of 488.03 ± 0.020 days and an AFC of 1063.48 ± 0.18 days in the Asturiana de los Valles breed. Similarly, Meneses et al. [48] found that the mean IC12 was 409 days (SD = 73) for the Avileña-Negra Ibérica and 453 days (SD = 102) for Retinta, which aligns closely with our findings.

### 3.2. Estimation of Variance Components and Heritability

Actually, routine genetic evaluations in the Retinta breed have been based on the BLUP methodology, utilizing only phenotypic and pedigree information. However, in recent years, genotyping has been introduced, enabling the application of genomic evaluation for economically important traits in this breed. The variance components and heritability ($h^{2}$) estimates are presented in Table 2.

The estimates of $h^{2}$ were 0.15 ± 0.008, 0.24 ± 0.015, 0.27 ± 0.012, and 0.20 ± 0.005 for AFC, IC12, ACI, and RE, respectively. Given these low-to-moderate heritability estimates, a relatively moderate genetic progress can be expected if selection is applied based on these traits. Our estimates of $h^{2}$ were in the range of values observed in beef cows of multiple breeds of different typologies worldwide by Cammack et al. [6] in their meta-analysis. Generally, the heritability of AFC is low to moderate in the literature. Smith et al. [49] and Martínez-Velázquez et al. [50] published heritability below 0.1. However, other authors reported higher values (0.235, [8]; 0.31, [7]; 0.27, [51]).

Our estimated heritability for IC12 was comparable to the 0.227 value reported by Cortés et al. [52], higher than those of other studies (0.02, [7]; 0.01, [53], and lower than the coefficient of 0.39 published by Veselá [54].

Heritability estimate for ACI in the present study is higher than the values usually reported in the literature. This trait is considered to be the one with the highest heritability among classic reproductive traits [48], since it is independent of the farmer’s decision to introduce the heifer in its first breeding batch (as is the case with AFC), and the animal has not yet had enough time to accumulate the impact of management and the environment in a significant number of births (as would be the case with ACI). In their review, Koots et al. [55] calculated an average heritability for CI from four published papers of 0.01 for multiparous cows and heifers, respectively. These differences between the different breeds could be explained by differences between genetic basis of each population and reaction to environmental conditions.

Nevertheless, none of these studies have employed RE as an indicator of female fertility. Genetic parameters of RE have been estimated previously in the Retinta breed using repeatability (Rep) and random regression models (RRM) and $h^{2}$ estimates were 0.3 using Rep and ranged from 0.24 to 0.51 with RRM [4]. Similarly, recent findings indicate $h^{2}$ values close to 0.25 in dairy goats [14] and horses [13].

Finally, it is worth highlighting the strong influence of the herd-season-year of birth interaction of the calf on all the reproductive traits of the cow (conditioning growth, the age at which puberty begins, the moment in which it is introduced into a breeding batch, etc.), its effect even being greater than the additive effect on AFC and RE.

### 3.3. Genome-Wide Association Studies

**Figure 1:**
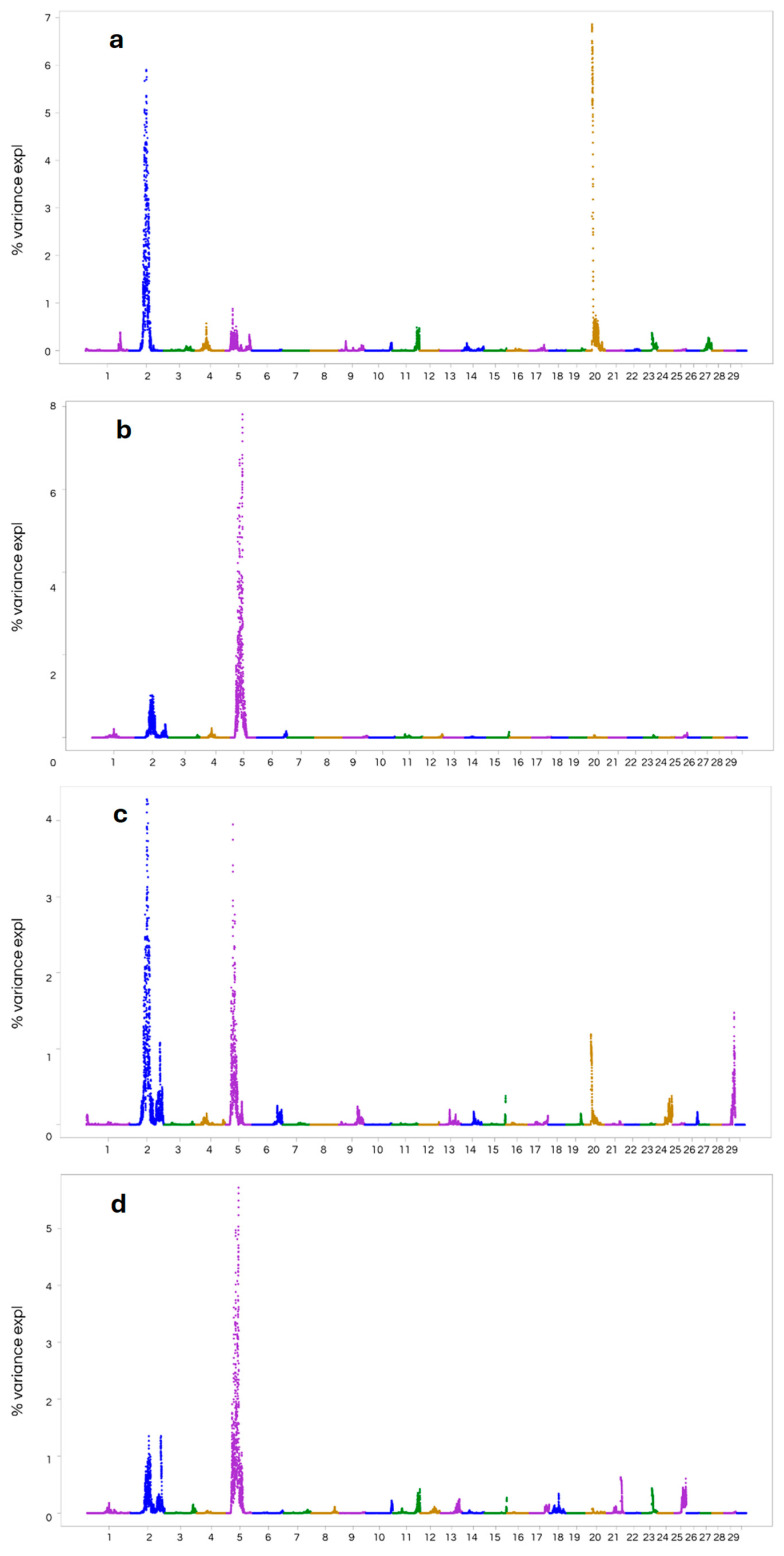
Manhattan plots representing the percentage of explained genetic variance by adjacent SNPs in 1 Mb window for the following traits: (**a**) Age at first calving (AFC); (**b**) Reproductive efficiency at the last calving (RE); (**c**) Interval between first and second calving (IC12); and (**d**) Average calving interval (ACI). Chromosomes are represented in different colors.

The wssGWAS found several genomic regions containing SNPs for the traits AFC, IC12, ACI, and RE on different chromosomes (Figure 1). The most significant signal for AFC was observed on BTA2, followed by BTA20 (Figure 1a), whereas the most prominent signals for IC12, ACI, and RE corresponded to BTA2 and BTA5 (Figure 1b–d).

Comprehensive information on the genomic windows identified for each trait that explained more than 1% of the additive genetic variance, together with the corresponding annotated genes within those regions, is presented in Table S1 of the Supplementary Material. For age at first calving (AFC), 17 windows surpassed the 1% threshold, with 16 located on chromosome 2, where the most informative window accounted for 5.75% of the trait variance. A single window on chromosome 20 was also identified, explaining 7.072% of the additive variance associated with AFC (Figure 1a). In the case of reproductive efficiency at the last calving (RE), 25 windows exceeded the 1% threshold, three on chromosome 2 and 22 on chromosome 5, the most significant of which explained up to 5.67% of the variance (Figure 1b). The interval between first and second calving (IC12) displayed the highest number of windows above this threshold, with a total of 29 distributed across four chromosomes: 14 on BTA2, 12 on BTA5, one on BTA20, and two on BTA29. The window explaining the largest proportion of variance for this trait was found on chromosome 2, accounting for 4.47% (Figure 1c). Finally, for average calving interval (ACI), all 25 windows identified were exclusively located on chromosome 5, with the most explanatory window accounting for 7.857% of the additive genetic variance (Figure 1d).

In GWAS models based on ssGBLUP it is common to present the percentage of additive genetic variance explained by the chromosome segment (generally 500 kb to 1 mb) instead of the level of significance of the association [13,56,57,58,59].

**Figure 2:**
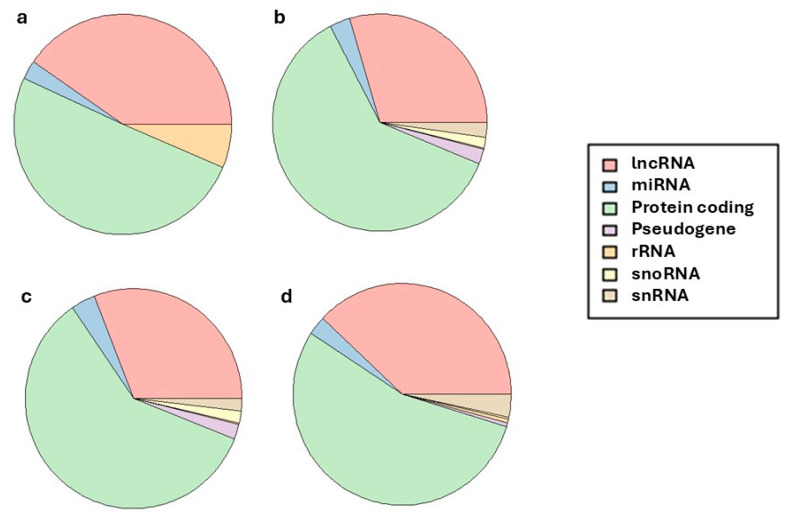
Pie chart of gene type found in the windows explaining more than 1% of the accumulative variance in the traits AFC (**a**), RE (**b**), IC12 (**c**) and ACI (**d**).

Of all the markers found, the majority (56.43%) corresponded to genes that generated coding proteins (Figure 2), 34.73% to lncRNA, and the remaining 8.83% were made up of other types of markers (miRNA, pseudogenes, rRNA, snoRNA and snRNA).

Long non-coding RNAs (lncRNAs) are a class of endogenous RNA transcripts longer than 200 nucleotides that do not encode proteins but are increasingly recognized as key regulators of gene expression at the epigenetic, transcriptional, and post-transcriptional levels [60]. lncRNAs exert their functions through diverse mechanisms, including chromatin remodeling, modulation of transcription factor activity, splicing regulation, and interactions with other RNA molecules. In livestock species, the study of lncRNAs has gained substantial interest due to their involvement in vital biological processes such as reproduction, growth, immune function, and metabolic regulation. Specifically in the context of reproduction, lncRNAs have been shown to modulate ovarian follicle development, oocyte maturation, spermatogenesis, and hormonal signaling pathways, thereby influencing fertility and reproductive efficiency [61]. Their tissue-specific expression patterns and regulatory versatility make them particularly valuable for elucidating the molecular mechanisms underlying complex reproductive traits.

### 3.4. Common Genes Between Traits

A total of 296 genes were found to be common across traits within the windows that explained more than 1% of the variance (Supplementary Table S2). Thirty-two genes were found to be common across traits AFC and IC12, 145 between RE and ACI, 2 between IC12 and ACI, of which 6 were common to AFC, RE, and IC12, and 111 between RE, IC12, and ACI. Among these, 21 genes were found to have direct evidence of metabolic and physiological aspects involved in reproductive issues (Table 3).

In a previous study, we identified 2 regions on BTA4 and BTA28 including some candidate genes associated with reproduction in cattle and other organisms for trait RE. These genes included *NRF1*, *SSMEM1*, and *CPA5* on BTA4, and *RYR2* and *ZP4* on BTA28 [4]. *NRF1* plays a role in cell signaling, protein biosynthesis, and proliferation of mitochondria, and a reduced expression of this gene is associated with embryonic lethality in mice and apoptosis of granulosa cells in goats [62,63]. *RYR2* and *ZP4* have also been associated with female fertility. For instance, the protein coded by *ZP4* can be found in the zona pellucida, essential for both oocytes and embryos. In fact, loss of function of *ZP4* impairs fertility in rabbits [64].

Otherwise, Reding et al. [65] identified several genes associated with AFC and IC12 in Bonsmara cattle. For example, they linked genes *PCDHGA*, *PCDHGB*, and *PCDHGC*, members of the protocadherin family that are critical for the development of ovaries and embryos [66,67], with IC12. In addition, 8 genes located on BTA7 and BTA13, i.e., ARAP3, *CLINT1*, *FCHSD1*, *LSM11*, *PLCB1, RELL2*, *SM11*, and *THG1L*, were common to both traits [65]. Of these, *PLCB1*, which encodes a phospholipase, is important for folliculogenesis [68].

However, these preliminary results were obtained with classical GWAS and a lower number of animals. Our new analysis, on the other hand, integrates pedigree, phenotypic, and genomic information from a larger population, increasing robustness and accuracy.

### 3.5. Cluster Analysis

The analysis of the molecular relationships between the products of protein-coding genes common across traits showed 282 connections, organized into 8 main clusters based on the number of interactions that occurred between them (Table 4).

**Figure 3:**
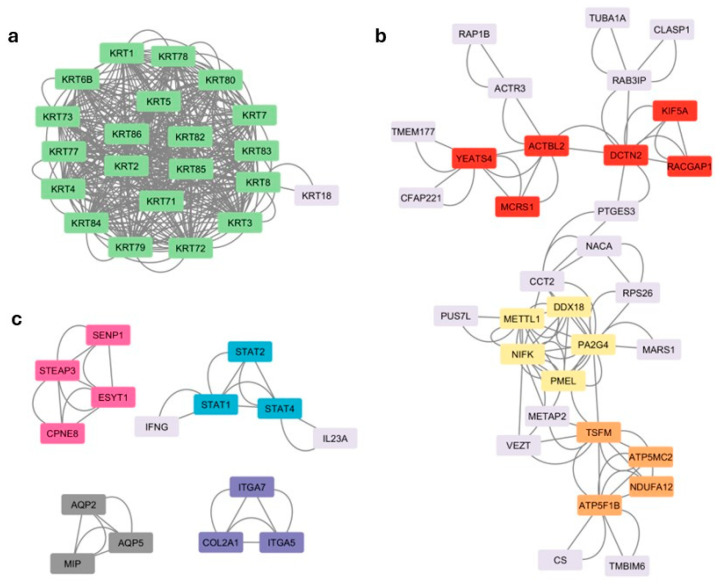
Representation of the results obtained after cluster analysis. (**a**) Cluster 1 (green); (**b**) Clusters 2 (red), 3 (yellow) and 4 (orange); (**c**) Clusters 5 (pink), 6 (blue), 7 (gray) and 8 (purple).

Of these, 5 are shown isolated (cluster 1, 20 connected proteins; cluster 5, 4 connected proteins; clusters 6, 7, and 8 with 3 connected proteins) while clusters 2, 3, and 4, formed by 6, 5, and 4 proteins, respectively, are interconnected with each other (Figure 3).

Cluster 1, the most prominent one, was constituted by 20 proteins from the Keratin family, which are constituents of the intermediate filaments (one of the main components of the cytoskeleton) with a key role in the organization, differentiation, and keratinization of epithelial cells. For instance, proteins encoded by *KRT18*, *KRT7*, and *KRT8*, expressed in uterus and ovary, are important for implantation and endometrium integrity in cattle [69]. This biological function could have an impact on the breeder’s recovery after giving birth, reducing the time to the next birth, with an important influence on traits IC12 and ACI. On the other hand, short calving intervals may decrease the date of the last birth. These genes explain 1.24%, 1.06%, and 1.38% of the variance for traits RE, IC12, and ACI, respectively.

Proteins from clusters 2 and 3 were associated with cell growth, differentiation, and organization, including processes such as regulation of cytoskeletal architecture, cell division, intracellular transport, and RNA processing, whereas cluster 4 included proteins involved in ATP production. All these processes are relevant for oocyte production and embryo development [70,71].

Similarly, clusters 5, 6, 7, and 8 included proteins involved in cell signaling, immune response, water transport across cell membranes, and organization of the extracellular matrix, respectively. For instance, cluster 6 includes the product of gene *STAT1*, which has a role in uterine immune response and embryo implantation, and has been associated with maternal-fetal tolerance [72]. *STAT1* belongs to a genomic window in BTA2 that explains the 2.9% and 2.4% of the variance of AFC and IC12, respectively, indicating the importance of this region. Furthermore, the protein encoded by *ITGA5* (cluster 8, with 1.196% of variance explained in RE and 1.073% in ACI) participates in cell adhesion and interactions with the extracellular matrix, processes that are key for embryo implantation and placental development [73].

In addition to genes grouped within functional clusters, several unclustered genes previously associated with reproductive traits in cattle were also identified. One such example is *ACVR1B* (activin receptor), a gene common to ACI, RE, and IC12, which regulates granulosa cell proliferation and steroidogenesis, thereby influencing ovulation and fertility [74]. Similarly, *AMHR2*, shared by RE and ACI, is a well-established marker of ovarian reserve and function, with known associations to age at puberty, ovulation rate, and fertility in cows [75]. *GDF11*, a member of the TGF-β family, is implicated in the regulation of folliculogenesis and ovarian development [76], while *PTGES3* plays a role in the biosynthesis of prostaglandins—molecules essential for ovulation, luteolysis, and implantation across various species including cattle [77]. The gene *SLC11A2*, involved in iron transport, is relevant given iron’s essential role in cell proliferation and ovarian function [78], and *SP1* has been shown to regulate the expression of genes involved in steroidogenesis and ovarian activity [79].

The genes *CDK2* and *CDK4*, both central to cell cycle progression during oocyte meiosis and granulosa cell proliferation [80], were identified within highly informative genomic windows for both RE (explaining up to 4.23% of variance) and ACI (up to 7.86%).

Furthermore, several genes associated with vitamin D metabolism were detected. *CYP27B1*, expressed in bovine ovaries, is involved in the synthesis of active vitamin D, which modulates both steroidogenesis and follicular function [81]. *VDR*, which encodes the vitamin D receptor, mediates the downstream effects of vitamin D in the regulation of ovarian function and steroid hormone production [82].

Other genes of reproductive relevance include *ERBB3*, which participates in growth factor signaling in the ovary and antral follicles and regulates granulosa cell proliferation and steroidogenesis [83], and *FRS2*, an essential mediator of FGF signaling involved in granulosa cell proliferation and folliculogenesis [84]. *HSD17B6* contributes to the conversion of androgens to estrogens, a critical step in both ovarian and testicular steroidogenesis [85]. *INSIG2* regulates cholesterol and lipid biosynthesis, fundamental precursors for steroid hormone production in gonadal tissues [86], while *NR4A1* is involved in the modulation of ovarian steroidogenesis and granulosa cell function [87]. Lastly, *IFNG* plays a crucial role in regulating the uterine immune environment, embryo implantation, and maternal–fetal tolerance, thereby influencing fertility and pregnancy outcomes [88].

The present study provides a comprehensive genomic characterization of female fertility traits in the Spanish Retinta beef cattle breed, managed under extensive conditions. By integrating pedigree, phenotypic, and genomic data through the weighted single-step GWAS methodology, we identified genomic regions and candidate genes associated with age at first calving, calving intervals, and reproductive efficiency. Several biologically relevant genes involved in ovarian function, hormone regulation, immune response, and cellular processes were highlighted, along with a notable representation of lncRNAs, suggesting regulatory complexity in the genetic control of fertility. These findings contribute to a more precise understanding of the molecular architecture underlying reproductive performance in extensively reared beef populations.

## 4. Conclusions

In this study, the identification of genomic regions with moderate-to-high contributions to additive genetic variance offers valuable tools for the development of genomic selection strategies in the Retinta breed. The incorporation of reproductive efficiency (RE) as a selection criterion, supported by its higher heritability and integrative nature, is particularly promising for improving fertility without compromising adaptation or maternal aptitude. Overall, the implementation of genomic-informed breeding programs based on the markers and genes identified herein may accelerate genetic progress, enhance productivity, and ensure the long-term viability of this locally adapted and economically important cattle population.

## Figures and Tables

**Table 1:** Descriptive Statistics for female fertility traits in the Retinta breed.

Trait	Mean	Minimum	Maximum	Std.Dev.	Coef.Var.
AFC	34.94 ± 0.032	17.03	48.9	6.72	19.24
IC12	15.12 ± 0.027	9.031	24.9	3.86	25.5
ACI	15.74 ± 0.019	9.03	25.5	3.30	20.9
RE	72.45 ± 0.105	22.0	100.0	21.74	30.0

AFC: age at first calving; IC12: interval between first and second calving; ACI: average calving interval; RE: reproductive efficiency. AFC, IC12, and ACI are expressed in months; RE is expressed as a percentage (%).

**Table 2:** Genetic parameters and heritability estimates (SE between parenthesis) for female fertility traits in the Retinta breed.

Trait	$\sigma_{a}^{2}$ (SE)	$\sigma_{hys}^{2}$(SE)	$\sigma_{e}^{2}$ (SE)	$h^{2}$ (SE)
AFC	6.29 (0.34)	15.90 (0.35)	21.01 (0.28)	0.15 (0.008)
IC12	3.65 (0.24)	2.49 (0.13)	8.90 (0.19)	0.24 (0.015)
ACI	2.91 (0.14)	1.45 (0.07)	6.26 (0.11)	0.27 (0.012)
RE	53.38 (2.16)	73.79 (1.81)	133.94 (1.71)	0.20 (0.005)

$\sigma_{a}^{2}$: additive genetic variance; $\sigma_{hys}^{2}$: herd-year-season variance $\sigma_{e}^{2}$: residual variance; $h^{2}$: heritability; SE: standard error; AFC: age at first calving; IC12: interval between first and second calving; ACI: average calving interval; RE: reproductive efficiency.

**Table 3:** Genes shared across different traits with previous evidence in bovines.

Traits	Nº of Genes	Candidate Gene	Gene Name
AFC, IC12	32	*INSIG2*	Insulin induced gene 2
		*STAT1*	Signal transducer and activator of transcription 1
RE, IC12, ACI	111	*ACVR1B*	Activin A receptor type 1B
		*FRS2*	Fibroblast growth factor receptor substrate 2
		*IFNG*	Interferon gamma
		*KRT18*	Keratin 18
		*KRT7*	Keratin 7
		*KRT8*	Keratin 8
		*NR4A1*	Nuclear receptor subfamily 4 group A member 1
		*VDR*	Vitamin D receptor
RE, ACI	145	*AMHR2*	Anti-Müllerian hormone receptor type 2
		*CDK2*	Cyclin-dependent kinase 2
		*CDK4*	Cyclin-dependent kinase 4
		*CYP27B1*	Cytochrome P450 family 27 subfamily B member 1
		*ERBB3*	Erb-B2 receptor tyrosine kinase 3
		*GDF11*	Growth differentiation factor 11
		*HSD17B6*	Hydroxysteroid 17-beta dehydrogenase 6
		*ITGA5*	Integrin subunit alpha 5
		*PTGES3*	Prostaglandin E synthase 3
		*SLC11A2*	Solute carrier family 11 member 2
		*SP1*	Sp1 transcription factor

**Table 4:** Clusters of proteins encoded by common genes and their functions.

Cluster	Description	Gene Symbol
1	Keratinization	*KRT78*, *KRT73*, *KRT84*, *KRT7*, *KRT86*, *KRT4*, *KRT6B*, *KRT77*, *KRT83*, *KRT71*, *KRT80*, *KRT2*, *KRT5*, *KRT1*, *KRT85*, *KRT3*, *KRT82*, *KRT79*, *KRT72*, *KRT8*
2	Cell cycle, Cytoskeletal organization, Chromatin remodeling	*MCRS1*, *ACTBL2*, *KIF5A*, *RACGAP1*, *YEATS4*, *DCTN2*
3	RNA processing, Pigmentation	*NIFK*, *PA2G4*, *PMEL*, *METTL1*, *DDX18*
4	ATP production	*TSFM*, *NDUFA12*, *ATP5MC2*, *ATP5F1B*
5	Protein regulation, Metal ion binding	*CPNE8*, *ESYT1*, *STEAP3*, *SENP1*
6	Cytokine-mediated signaling pathway	*STAT4*, *STAT2*, *STAT1*
7	Water channel activity	*MIP*, *AQP2*, *AQP5*
8	Cell adhesion, Extracellular matrix organization	*ITGA7*, *COL2A1*, *ITGA5*

## Data Availability

The dataset employed in this study are property of the National Association of Breeders of Selected Retinta Cattle (ACRE) and were provided for scientific purposes under a specific collaboration arrangement. The dataset can be made available for scientific purposes to other authors by the ACRE technical department, under reasonable request.

## Supplementary Materials

The following supporting information can be downloaded at: https://www.mdpi.com/article/10.3390/ani15182665/s1, Figure S1: Manhattan plot for AFC; Figure S2: Manhattan plot for IC12; Figure S3: Manhattan plot for ACI; Figure S4: Manhattan plot for RE; Figure S5: Pie chart of gene type found in the windows explaining more than 1% of the accumulative variance in the studied traits; Figure S6: Representation of the results obtained after cluster analysis; Table S1: Genomic windows explaining more than 1% of additive variance for the studied traits; Table S2: Common genes across studied traits.
