# Supplementary figures and images for: Weighted Single-Step GWAS Reveals Genomic Regions Associated with Female Fertility in the Spanish Retinta Beef Cattle
Source: Animals (Basel). 2025 Sep 11;15(18):2665. doi: 10.3390/ani15182665 (PMC12466598; doi:10.3390/ani15182665)

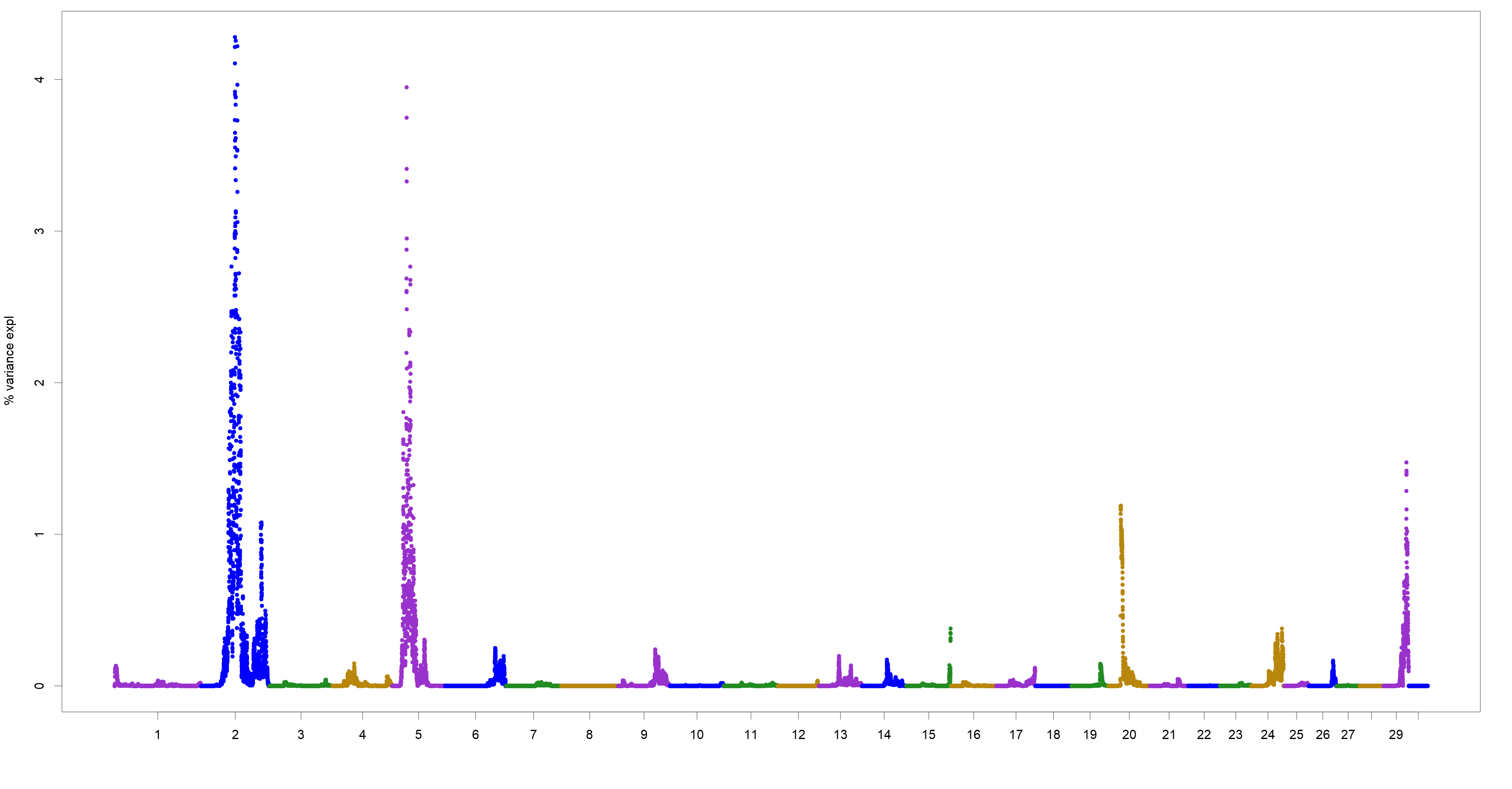

Supplement: Supplementary file 1 [file animals-15-02665-s001.zip › Figure S2.png]

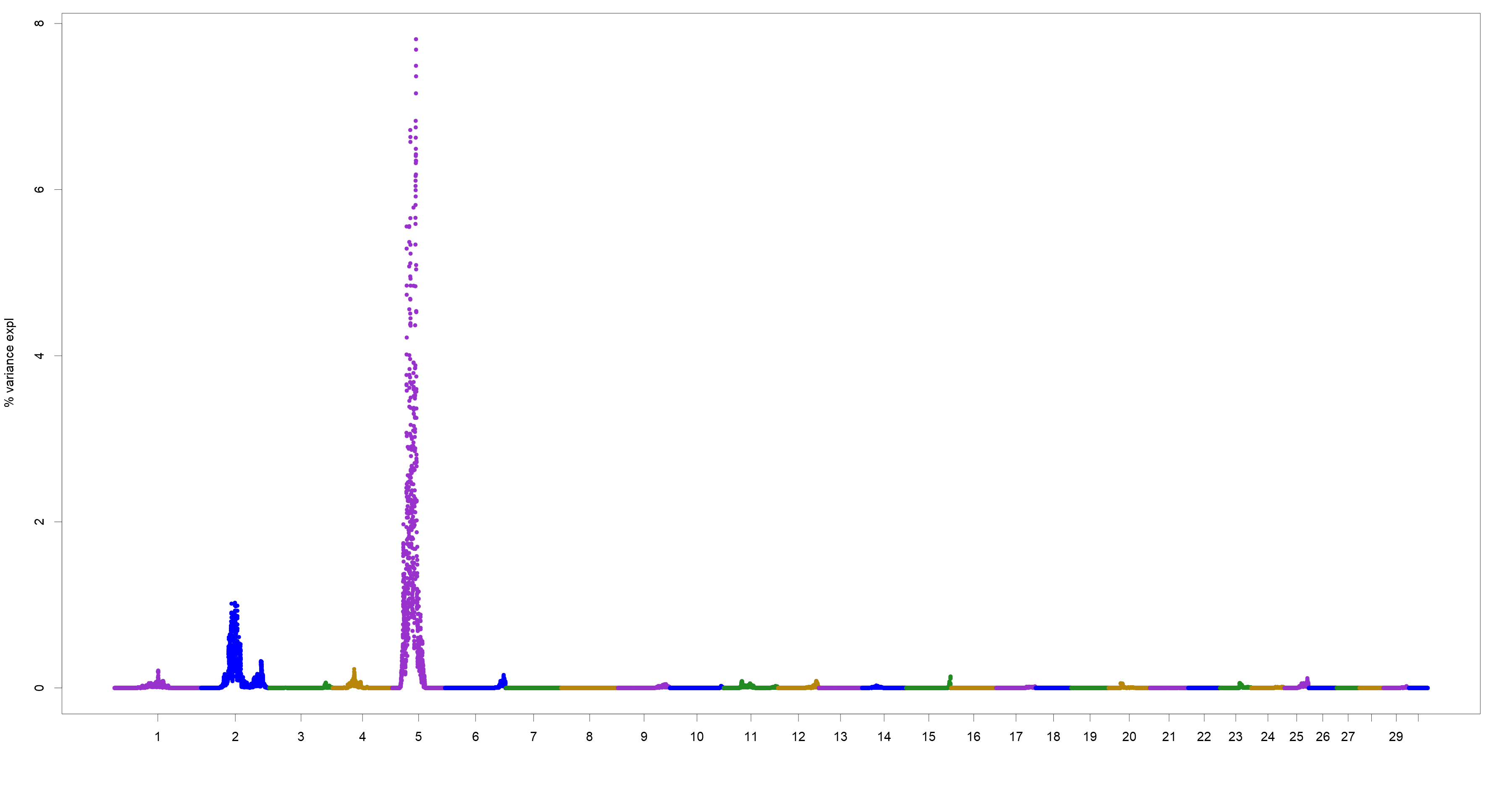

Supplement: Supplementary file 1 [file animals-15-02665-s001.zip › Figure S3.png]

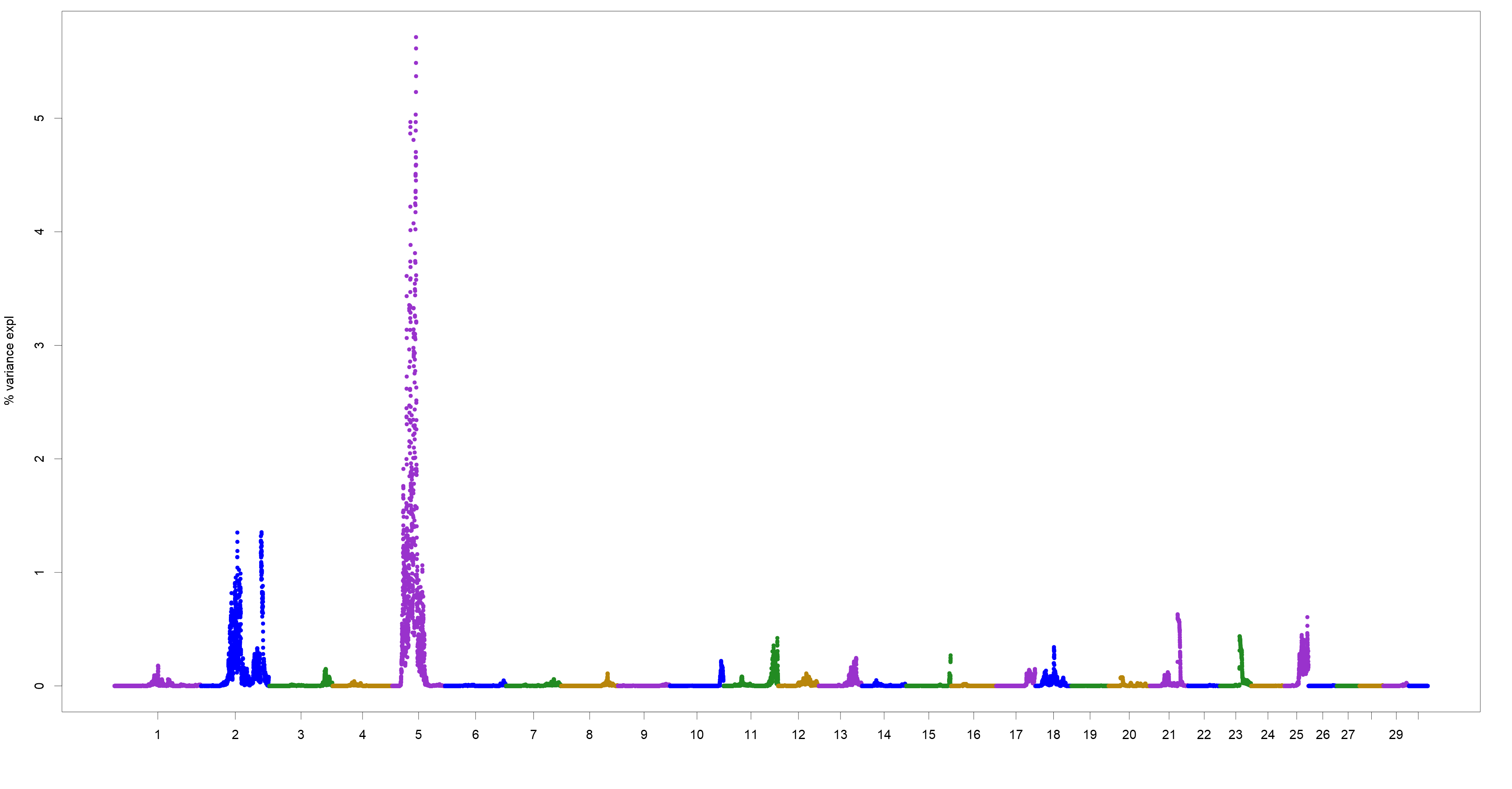

Supplement: Supplementary file 1 [file animals-15-02665-s001.zip › Figure S4.png]

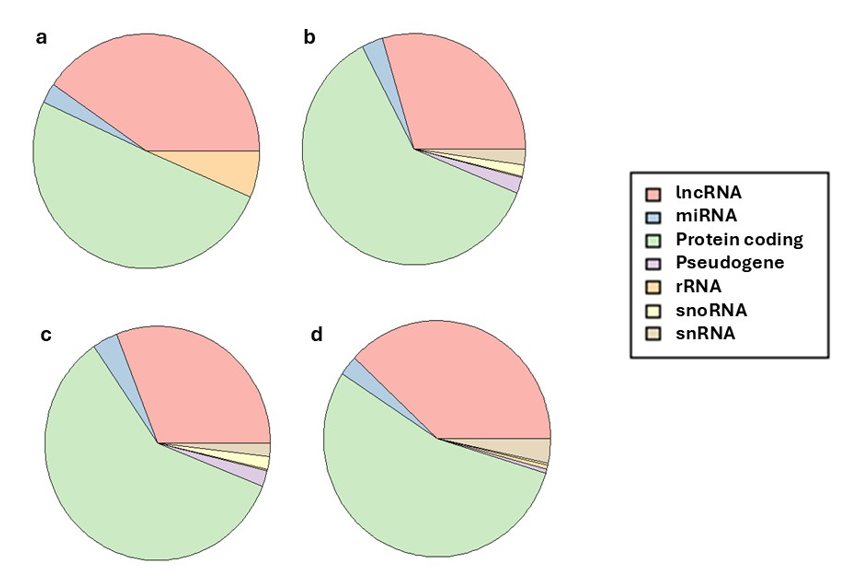

Supplement: Supplementary file 1 [file animals-15-02665-s001.zip › Figure S5.png]

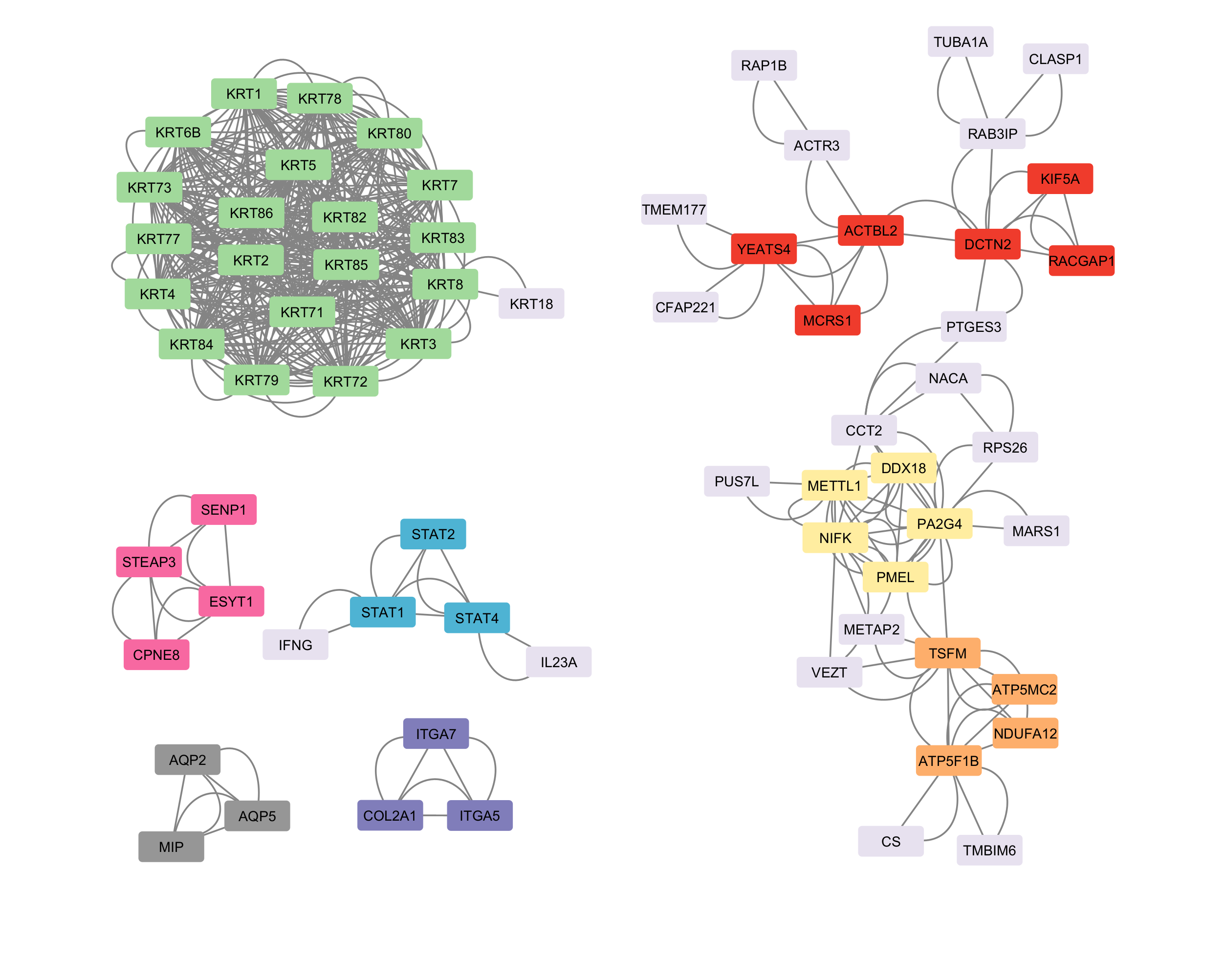

Supplement: Supplementary file 1 [file animals-15-02665-s001.zip › Figure S6.png]

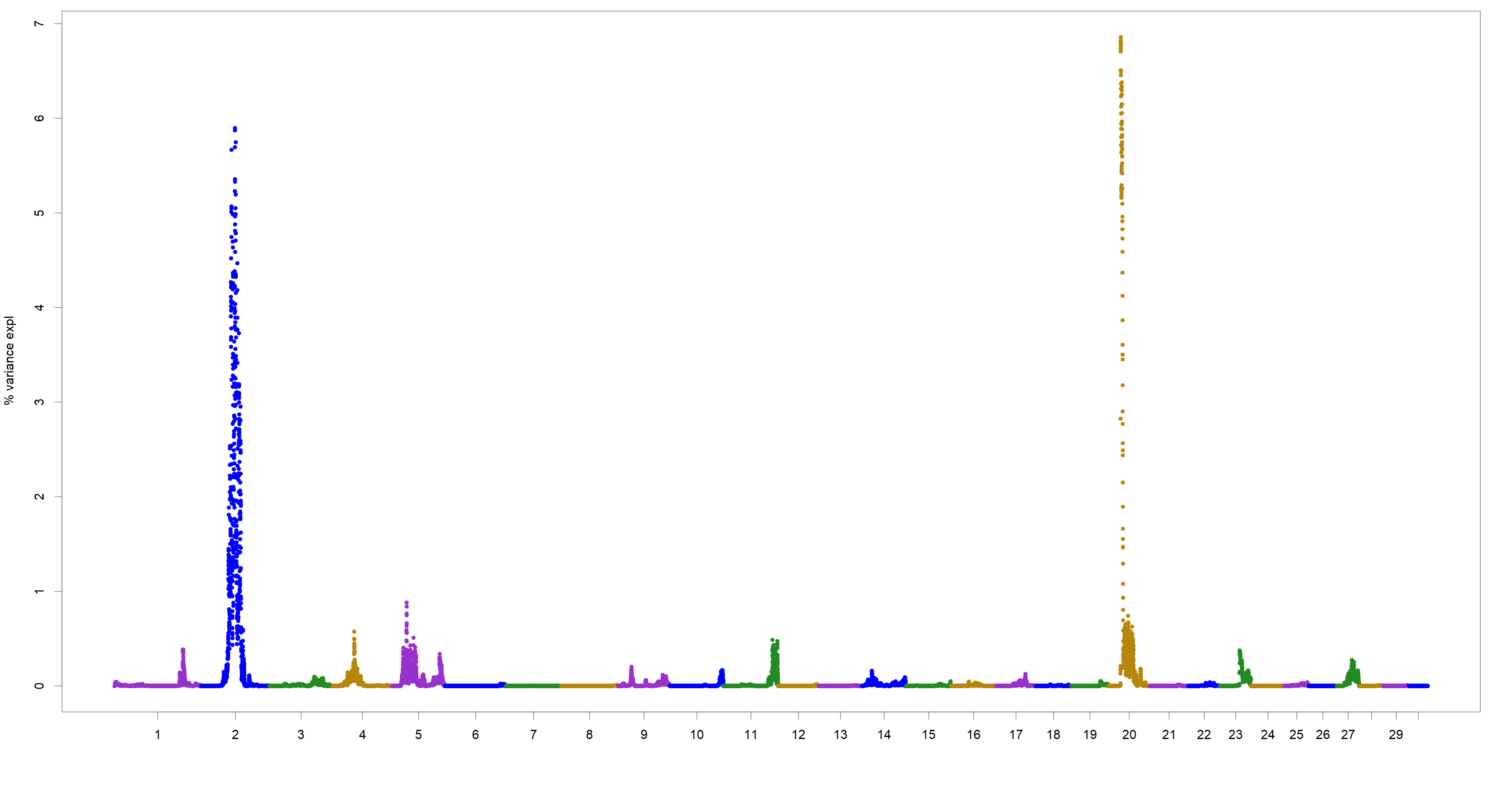

Supplement: Supplementary file 1 [file animals-15-02665-s001.zip › Figure S1.png]
